# Comparative outcomes of unilateral biportal endoscopic lumbar intervertebral discectomy with and without annulus fibrosus suture in lumbar disc herniation: a retrospective analysis

**DOI:** 10.3389/fsurg.2025.1521892

**Published:** 2025-04-29

**Authors:** Wei Zhou, Mohamed Lamin Bangura, Qianlong Gong, Rong Zhang, Teng Zeng, Qi Fei, Tadiwa Chiedza Chirima, Sy-Trung Tran, Yutian Qiu, Huasong Luo

**Affiliations:** ^1^ Department of Orthopedics, The First People’s Hospital of Jingzhou, The First Affiliated Hospital of Yangtze University, Jingzhou, Hubei, China; ^2^ Yangtze University Health Centre, Jingzhou, Hubei, China

**Keywords:** lumbar disc herniation, unilateral biportal endoscopic lumbar discectomy, spinal nerve root decompression, fiber ring suture, minimally invasive surgery

## Abstract

**Background:**

Advancements in minimally invasive spine surgery have markedly enhanced patient outcomes in the management of lumbar intervertebral disc herniation and degenerative disorders. The Unilateral Biportal Endoscopic Interlaminar Lumbar Intervertebral Discectomy and spinal nerve decompression are prominent of these methods. This method is based on the principles established by several endoscopic spine techniques, which are lauded for their limited invasiveness, less trauma, and expedited recovery periods in contrast to conventional open operations.

**Methods:**

177 patients treated with Unilateral Biportal Endoscopic Transforaminal Lumbar Intervertebral Discectomy for lumbar disc herniation were selected and assigned into Sutured (39 patients) and Un-sutured groups (138 patients). Different variables, including clinical outcomes and estimated cost, were evaluated using IBM SPSS 27.0.1 with a *p*-value < 0.05 considered statistically significant.

**Results:**

The study identified disparities in clinical outcomes, such as reoperation problems, surgery durations, and projected costs between the two groups. Reoperation rates were lower in the sutured group. Un-sutured patients had a shorter surgery time. Both groups had comparable Visual Analog Scale (VAS) and Japanese Orthopaedic Association (JOA) scores. Both groups have similar Body Mass Indexes (BMIs) throughout hospitalization. The two groups had equal discharge satisfaction scores. There is not much variation in surgical bleeding across groups. Follow-up times were identical for both groups (26.46 ± 2.01 for the sutured group and 26.83 ± 2.68 for the un-sutured group). The two groups showed a slight difference in estimated costs, with the sutured group averaging RMB 29,234.78 ± 5,265.83, compared to RMB 22,311.10 ± 3,527.00 for the un-sutured group.

**Conclusion:**

Annulus fibrosus suturing during minimally invasive lumbar disc surgery may increase time and expense and reduce the risk of recurrent herniation and reoperation. Sutured and non-sutured techniques have equal clinical results and low intraoperative blood loss, making them feasible alternatives depending on the situation and patient demands.

## Introduction

Recently, with the introduction of minimally invasive spine surgery as an alternative treatment for lumbar intervertebral disc herniation and degenerative diseases, the cure rates and outcomes have increased over the last few years. The Unilateral Biportal Endoscopic (UBE) Lumbar Intervertebral Discectomy and nerve root decompression in a distinctive way. Improved upon the groundwork laid by numerous endoscopic spine procedures celebrated for a less invasive, less traumatic, and quicker recovery than traditional open surgeries (1–3).

Among all, percutaneous endoscopic lumbar discectomy (PELD) is a mini-invasive technique that has promising short-term outcomes, in particular, the transiliac approach to intervertebral endoscopic discectomy for L5/S1 intervertebral disc herniation (1). However, comparative studies have demonstrated that microendoscopic discectomy and microdiscectomy do not have this characteristic that showed a clear and significant superiority of percutaneous endoscopic transforaminal endoscopic discectomy (PETD) over these methods regarding clinical outcome with less surgical trauma or fast recovery (2, 3). In recent years, minimally invasive transforaminal lumbar interbody fusion (MIS-TLIF) and its percutaneous variant have also been popularized. These procedures use intervertebral foramen for decompression and fusion without removing the bone structure, preserving vertebral stability to favor faster physical recovery (3). This further illustrates a trend toward ultra-minimally invasive surgery with the development of full-endoscopic assisted lumbar decompressive surgery. These outpatient procedures have consistently demonstrated a low complication rate and symptomatic improvement, emerging as therapeutic options for patients with lumbar stenosis or other related pathologies (4, 5).

Although many studies have shown the beneficial effect of these minimally invasive procedures, little or no data are available regarding annulus fibrosus suturing and its advantages over not suturing. Hence, focusing on the potential advantages of annulus fibrosus suture concerning unilateral bi-portal endoscopic transforaminal lumbar intervertebral discectomy after spinal nerve decompression represents a valuable effort to explore and address the complexities of this application within minimally invasive spine surgery expansions.

This study, therefore, aims to quantify the financial consequences resulting from suturing compared with not suturing. In addition, it will also provide some statistical evidence for spine surgeons to determine whether or not the annulus fibrosus should be sutured after nerve decompression in the treatment of lumbar disc herniation.

## Materials and methods

Our institution's ethics committee approved this study for medical research, and informed consent was obtained from all participants. Follow-up evaluations were conducted either during outpatient visits or via phone calls. Although the department began performing this surgical procedure in January 2020, to minimize the impact of the learning curve, we selected patients who underwent surgery for lumbar disc herniation starting in March 2021. All patients included in the study had a minimum follow-up period of 24 months. 177 patients treated with Unilateral Biportal Endoscopic Inter-Laminar Lumbar Intervertebral Discectomy for lumbar disc herniation were selected and assigned into Sutured (39 patients) and Un-sutured groups (138 patients). Different variables, including clinical outcomes and estimated cost, were evaluated using IBM SPSS 27.0.1 with a *p*-value < 0.05 considered statistically significant. The patients' characteristics of each patient of both groups are summarized in Table 1.

**Table 1:** The outcomes of participants.

Serial no	Variables	Sutured Group (*N* = 39)	Un-sutured Group (*N* = 138)	*p*-value
1	Age (years)	57.98 ± 13.65	59.26 ± 12.16	0.571
2	Gender	Total (%)	Total (%)	
	Male	13 (33.3)	69 (50)	0.491
	Female	26 (66.7)	69 (50)	0.42
3	BMI	24.21 ± 3.26	24.53 ± 3.20	0.586
4	Surgical duration(minutes)	125.51 ± 39.33	96.4 ± 25.25	<0.001
5	Follow-up period (months)	26.46 ± 2.01	26.83 ± 2.68	
6	Hospitalization period (days)	9.17 ± 1.93	9.03 ± 2.78	0.751
7	Average estimated cost (¥)	29,234.78 ± 5,265.83	22,311.10 ± 3,527.00	<0.001
8	Leg VAS score			
	Pre-operation	6.4 ± 1.02	6.29 ± 1.04	0.546
	At discharge	2.59 ± 0.55	2.6 ± 0.58	0.696
	6 months post-operation	1.00 ± 0.89	1.05 ± 0.76	0.688
	12 months post-operation	0.26 ± 0.44	0.30 ± 0.49	0.284
9	Back pain VAS score			
	Pre-operation	5.23 ± 0.74	5.35 ± 0.73	0.380
	At discharge	2.59 ± 0.54	2.63 ± 0.58	0.696
	6 months post-operation	1.00 ± 0.79	1.05 ± 0.76	0.270
	12 months post-operation	0.26 ± 0.44	0.30 ± 0.49	0.584
10	Bleeding (ml)	29.49 ± 13.76	29.64 ± 19.91	0.965
11	Reoperated	Total (%)	Total (%)	
12	Yes	1 (2.6)	7 (5.1)	
	No	38 (97.4)	131 (94.9)	
13	Complication	Total (%)	Total (%)	
	Recurrence	0 (0)	5 (3.6)	<0.01
	Compression due to hematoma	1 (2.6)	2 (1.4)	
14	Patients’ satisfaction	Total (%)	Total (%)	
	Highly satisfy	37 (94.9)	117 (84.9)	
	Satisfy	2 (5.1)	21 (15.5)	0.326
15	Mean JOA score Pre-operation At discharge	9.92 ± 2.59 20.13 ± 2.70	9.49 ± 2.36 22.49 ± 3.15	<0.001

The inclusion criteria were: (1) Patients treated for 1-level lumbar disc herniation at L3/L4, L4/L5, and L5/S1. (2) Patients with a minimum follow-up of two years. (3) Patients with primary lumbar disc herniation, (4) Patients whose signs and symptoms failed to relieve after conservative treatment for 3 months and above. (5) Patients whose disc herniation was confirmed by physical examination and radiological exams (MRI&CT).

Exclusion criteria: (1) Patients treated for 2-level disc herniation. (2) Patients with reoccurrence of disc herniation. (3) Patients who could not complete the minimum follow-up period. (4) Patients with lumbar stenosis other than herniation. (5) Patients with segmental instability.

### Surgical procedure

After successful anesthesia, the patient was placed in a prone position on a radiolucent table, and the appropriate lumbar gap was identified and marked using a C-arm fluoroscopy. Under C-arm fluoroscopy, the lumbar gap was positioned 1 cm above and below the midline, projecting onto the inner edge of the pedicle. Routine disinfection was performed, and drapes were applied. Using a #11 scalpel blade, two incisions were made, and the skin at the upper and lower positioning points was longitudinally widened by about 1 cm with a scissor. One channel was designated for endoscopy and the other for surgical operations.

**Figure 1:**
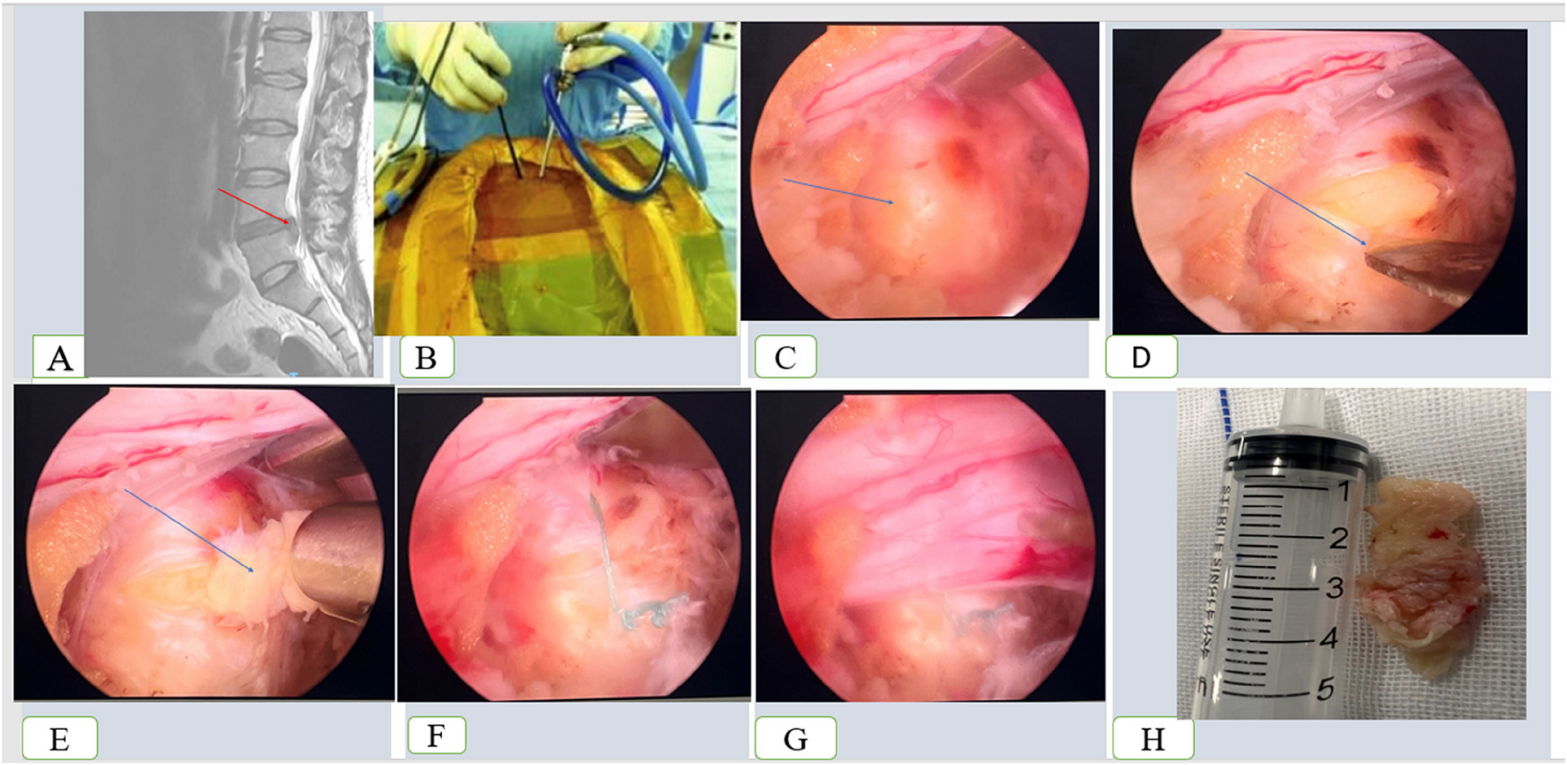
Procedures of the unilateral biportal endoscopic lumbar discectomy on a 54 years female treated for L4/L5 lumbar disc herniation. **(A)** Preoperative MRI, **(B)** point of insertion of the endoscopy and the associated instrument, **(C)** arrow pointing to the herniated disc, **(D)** process of incision of the herniated disc, **(E)** process of removing the nucleus fibrosis, **(F)** sutured annulus ring, **(G)** released nerve, and **(H)** nucleus fibrosis.

Using C-arm fluoroscopy, a disposable radiofrequency plasma knife was used to establish a channel at the inner edge of the appropriate vertebral plate and its inferior articular process. The imaging system was adjusted, and the area was observed under a microscope, with continuous rinsing using physiological saline. A disposable radiofrequency plasma knife was employed for electrocoagulation to stop bleeding during the procedure. The working channel was rotated, and a grinding drill and vertebral plate biting forceps were used to partially resect the bony vertebrae until the ligamentum flavum was exposed, as shown in Figure 1.

A blunt nerve hook was then used to separate the lower surface of the ligamentum flavum vertebral plate until it approached the lateral recess. Vertebral plate forceps were employed to expand the contralateral recess of the spinal canal for decompression. After removing the ligamentum flavum, the nerve root was explored, released and revealed the protruding intervertebral disc. The #11 scalpel blade was used to incise the protruding annulus fibrosus, a nucleus pulposus forceps was then used to remove the protruding intervertebral disc, and the annulus fibrosus was sutured using a fibrous ring stapler. For the un-sutured group, the annulus fibrosus ring was left open. The nerve root was checked for relaxation, and the dural sac was observed.

Finally, the working and endoscopic channels were withdrawn, a draining tube was placed, and the incision was sutured. The surgery was completed successfully, the anesthesia was satisfactory, and the patient was safely returned to the ward.

### Suturing techniques

**Figure 2:**
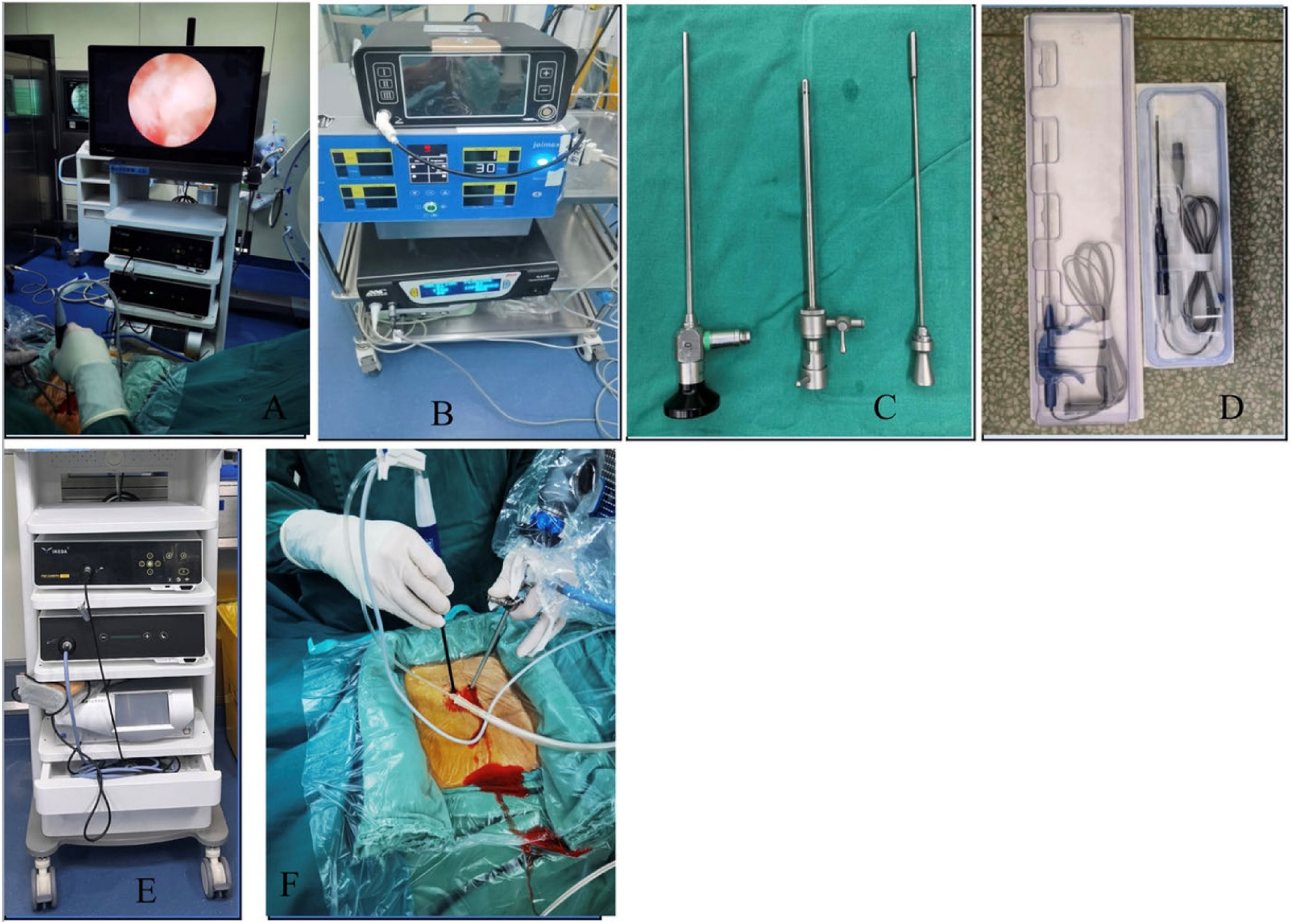
IKEDA FHD endoscopic camera system and other devices. **(A)** Endoscopic camera system with monitor **(B)** Power cutting devise **(C)** Arthroscopic lenses and sheath **(D)** Plasma knife **(E)** Endoscopic camera system with monitor without the monitor **(F)** Point of insertion of the endoscopy and the associated instrument.

Suturing was done with the Disposable Fiber Ring Suture Device, and the IKEDA FHD endoscopic camera System (Figure 2) was used to visualize the surgical field. The imaging light source system could provide a high-definition surgical site visualization consisting of the light source, the imaging system, and the display. The fiber optics were subjected to a sterilization system before use to maintain the aseptic conditions.

**Figure 3:**
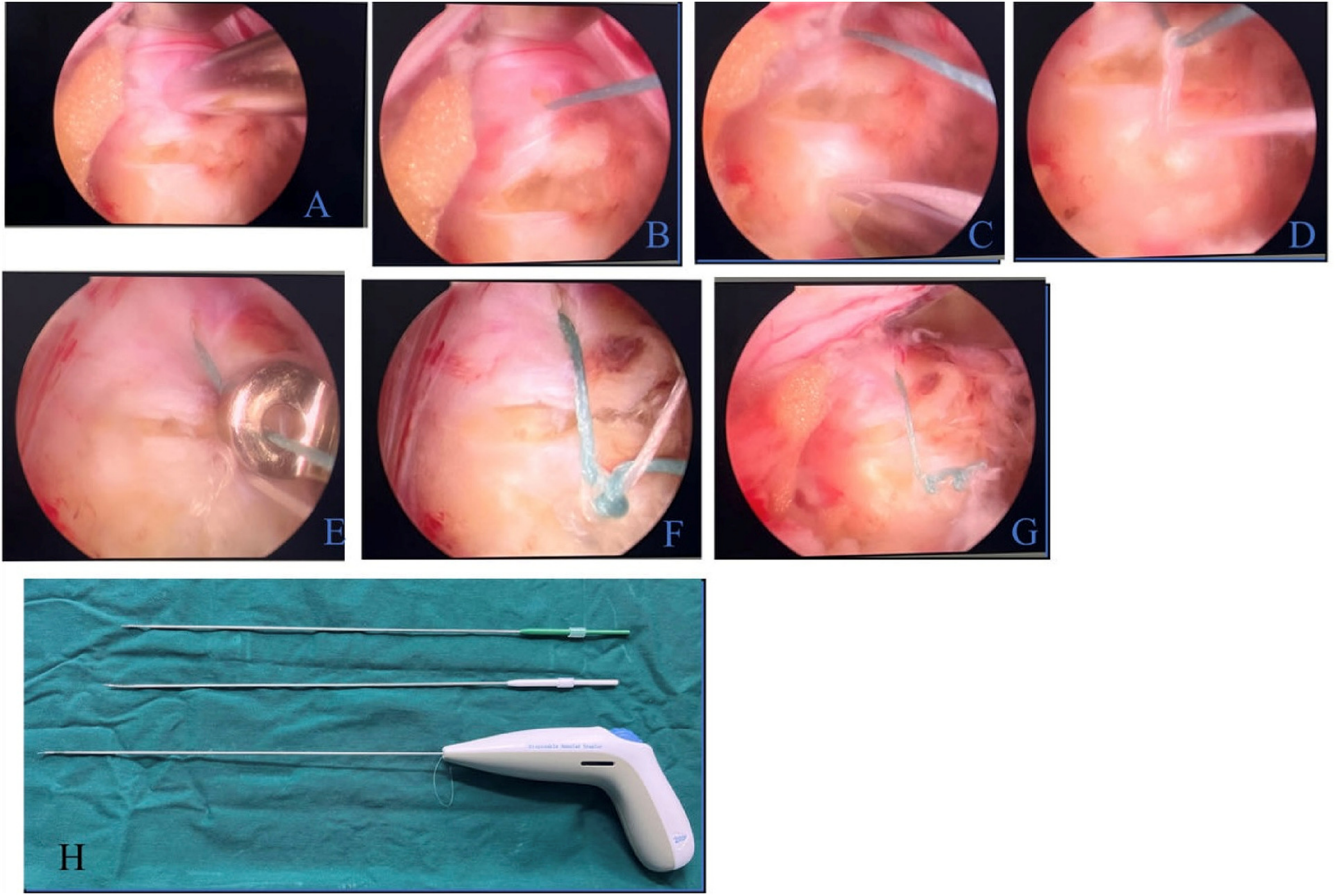
Suturing procedure. **(A)** Placement of the stapler **(B)** Exposed suture after incision **(C)** Insertion at the opposite side of the fibrous ring **(D)** and Knotting of Suture **(E)** Application of knot pusher **(F)** Knotted suture **(G)** Cut suture **(H)** Disposable fiber ring suture device.

The suturing procedure (Figure 3) was performed as follows:

Placement of Stapler: The Disposable Fiber Ring Suture Device was grasped and inserted vertically from one side of the fibrous ring incision, allowing for precise positioning.
Forward Knob Rotation: The knob was rotated forward as far as possible, enabling the device to lock around the incision site and hold the suture.
Button Adjustment: The button was advanced until the stopping point was reached to bring the suture into the proper position.
Knob Rotation (Backward): The knob rotated fully in the backward direction until it reached the endpoint, completing a proximity loop around the incision.

Removal of the Stapler and Tightening of the Knot: The stapler was removed, and a knot pusher was applied to the suture and tightened. The suture was cut with scissors, which completed the stitching process.

This strategy provides a low-cost, efficient, low-impact technique that enables precise suturing and optimum visualization using the IKEDA FHD Camera System.

### Post-operative treatment

All patients were administered intravenous antibiotics postoperatively to prevent infection, as well as analgesics for pain management. The drainage tube was withdrawn on the second day following the operation. Patients were instructed to bed rest for 3 weeks, supported with a waist brace for over 1 month.

### Clinical outcomes

The visual analog (VAS) scale was used to analyze back and leg pain before and after the surgery. The Japanese Orthopedics Association (JOA) score, developed to assess patients' clinical symptoms with herniated lumbar disc, was used to evaluate the improvement following the operation. A minimum score of −6 indicates the worst symptoms. The higher the score, the more regular the patient's condition.

### Sample characteristics

The normality of distribution of both age and BMI was evaluated using the Shapiro–Wilk test between the two groups. It showed that the BMI was approximately normally distributed in the sutured group (*p* > 0.05), but this was not the case in the un-sutured group. The age was also approximately distributed between the two groups with a *p*-value of 0.145 and 0.029 between the sutured and un-sutured, respectively, as shown in Table 2. A visual inspection of their standard Q-Q plots, histograms, and box plots confirmed that they are approximately distributed. An independent-sample *t*-test was also conducted on the age, affected level, hospitalization period, BMI, and the total estimated cost for both treatment options.

**Table 2:** Test for normality.

Variables	Groups	Shapiro-Wilk *p*-value
BMI	Sutured	0.370
BMI	Un-sutured	0.020
Age	Sutured	0.145
Age	Un-sutured	0.029

## Results

The sutured group had 39 patients, 13 males, and 26 females, with a mean age of 57.98 ± 13.65 years. The un-sutured group had 138 patients, 69 males and 69 females, with a mean age of 59.26 ± 12.16. The table in the Supplementary File summarizes detailed patient characteristics for both the sutured and un-sutured groups.

The study found differences in clinical outcomes, such as complications leading to reoperation, surgical durations, and the estimated cost, between the two groups. The sutured group had a lower reoperation rate than the un-sutured group and a longer operation time. Both groups had comparative outcomes in the VAS and JOA scores.

The BMI of both groups is similar, just to the hospitalization period. The two groups also shared similar satisfaction ratings at discharge. There were not many differences in operative bleeding in both groups. Both groups shared similar follow-up periods (26.46 ± 2.01 for the sutured group and 26.83 ± 2.68 for the un-sutured group). The estimated cost for both groups was recorded, with the sutured group averaging RMB 29,234.78 ± 5,265.83 and the un-sutured group recorded RMB 22,311.10 ± 3,527.00. The analysis of the result is recorded in Table 1.

## Discussion

This study will evaluate the effectiveness and safety of suturing annulus fibrosus compared with the un-suture counterpart in a unilateral bipolar endoscopic lumbar intervertebral discectomy during lumber spinal nerve decompression. Despite the similarities between both groups, the most notable differences identified by our study were surgical duration, complication rates, reoperation rates, and estimated total cost.

One of the most striking findings was the substantial variation in the surgical duration between the two groups. Both groups showed a statistically significant difference in surgical duration, as the sutured group lasted longer (125.51 ± 39.33 min) than the un-sutured group 96.40 ± 25.25 (*p* < 0.001), primarily due to additional time needed to close the annulus fibrosus. Though only a few studies directly compared these two methods, many other investigations look at minimally invasive vs. traditional open surgical outcomes (6–10). For example, Yao et al. compared minimally invasive transforaminal lumbar interbody fusion (MIS-TLIF), microendoscopic discectomy (MED), and percutaneous endoscopic lumbar discectomy (PELD). The total operation time was significantly longer with the MIS-TLIF, mainly because of the time needed to insert the implant (intervertebral fusion) (11). Similarly, Huang et al. (3), comparing interlaminar endoscopic lumbar discectomy (IELD) and transforaminal endoscopic lumbar discectomy (TLED), found that the procedures had similar average surgical duration. Our recorded shorter surgical duration aligns with other minimally invasive procedures previously compared to traditional open surgeries (such as anterior lumbar interbody fusion, which lasts significantly longer) (7–10).

Conversely, complication rates, including recurrent disc herniation, differed between the two groups. The group without sutures had more sessions of re-herniation, which led to a much higher re-operation rate. Liu et al. (2), in comparing minimally invasive techniques for treating lumbar disc herniation, reported 5 reoperations secondary to recurrent herniations. Takebayashi et al. (8) also documented five cases of recurrent herniations in a study comparing interlaminar and transforaminal approaches for full-endoscopic discectomy of an L4/L5 disc herniation. On the other hand, most previous reports do not especially indicate suture for annulus fibrosus during recounting of the recurrence (6, 7, 12). However, the lower recurrence rate in the sutured group could be attributed to the reinforced annulus fibrosus with sutures.

The overall cost of the non-sutured group was less than that of the sutured group, as demonstrated by our cost analysis (*p* < 0.001), and was primarily due to the additional staple used in suturing. While studies comparing the cost of different minimally invasive procedures are limited, Parker et al. (12) reported an increased cost of MIS-TLIF over open TLIF. However, the difference was more minor.

This study did not identify a statistically significant difference in intraoperative bleeding between the two groups. This finding is unsurprising, given that a similar bleeding outcome has been reported in minimally invasive surgeries (13–15). On the other hand, previous research has shown that intraoperative blood loss in traditional open surgery groups is more significant compared to minimally invasive techniques (13).

The VAS scores of both groups in this study consistently improved, with significant clinical improvements in pain. Except for reoperated cases, the patients had a gradual amelioration of symptoms. Similarly, no significant differences were noted when the Japanese Orthopaedics Association (JOA) scores between the two groups were compared, indicating that both approaches are practical overall. Earlier studies have reported similar results, regardless of whether a minimally invasive or open surgery was employed, indicating the common benefits of methods in managing lumber disc herniation (16–21).

## Strengths and limitations

Our detailed comparison of each annulus fibrosus closure technique makes this study a direct comparative analysis of two distinct sutured and non-sutured methods in minimally invasive spinal procedures. This retrospective analysis uses accurate clinical data, which better documents everyday, real-life practices and outcomes in day-to-day clinical practice. This study focuses on a relevant clinical endpoint as the reoperation rate is compared between sutured and non-sutured groups with the promise of benefit by using annulus fibrosus suturing to decrease recurrence rates. Adding cost analysis will help clinicians and hospital administrators compare the financial impact of sutured vs. non-sutured techniques.

Given its retrospective nature, there is potential for selection bias and no controlled environment that a randomized trial would provide. Even with statistical adjustments, the outcomes observed may be influenced by confounding factors. Since the study only evaluated less than a 5-year follow-up period, the long-term consequences, such as recurrence and reoperation rates after 5 years attributable to the suturing technique, may not have been covered. Without randomization, unmeasured factors could have played a role in what led a patient to either select the suture or non-suture procedure (e.g., a surgeon's preference or specific spinal pathology in each patient). Because the study was performed at a single institution, this could limit the external validity, and it can be difficult to generalize the results to other settings or regions with varied surgical practices. Confirmation of the efficacy of the two methods requires a randomized, multicenter, and long-time control trial.

Discectomy in a degenerative disc resulting from compromised disc integrity may have long-term consequences. The cartilaginous endplate is crucial for nutrition transport and mechanical load distribution. If its structure alters, the disc may deteriorate more rapidly (22). Peripheral ring apophyseal fractures (PRAF) may occur when the bone at the vertebral endplate-disc junction is subjected to excessive stress or fractured due to an accident (23). Numerous studies have indicated that alterations in the morphology of the endplates, including reduced thickness and variations in diameters from anterior to posterior, are risk factors for lumbar disc degeneration (22, 23). These factors are not included in this study and deserve to be considered in future studies to provide enhanced treatment techniques.

## Conclusion

Conclusively, a comparison of the results of this study showed that whereas annuls fibrous suturing during minimally invasive lumbar disc operations prolongs the surgical period and heightens cost, it lowers both the risk of recurrence herniation and the need for reoperation. The sutured group showed a better JAO score at discharge than the un-sutured group. The clinical outcome of the sutured and non-sutured methods seemed relatively similar, with slight variations in blood loss; thus, both approaches are considered acceptable options for lumber discectomy.

## Data Availability

The original contributions presented in the study are included in the article/Supplementary Material, further inquiries can be directed to the corresponding author.
