# Supplementary material for: Comparative outcomes of unilateral biportal endoscopic lumbar intervertebral discectomy with and without annulus fibrosus suture in lumbar disc herniation: a retrospective analysis
Source: Front Surg. 2025 Apr 29;12:1521892. doi: 10.3389/fsurg.2025.1521892 (PMC12069353; doi:10.3389/fsurg.2025.1521892)
Supplement: Supplementary file 1 [file Table1.docx]

| DATA FOR UNILATERAL BI-PORTALL ENDOSCOPIC LUMBER INTERVERTEBRAL DISC RESECTION AND DECOMPRESSION | | | | | | | | | | | | | | | | |  |  |  |  |  |  |  |  |
| --- | --- | --- | --- | --- | --- | --- | --- | --- | --- | --- | --- | --- | --- | --- | --- | --- | --- | --- | --- | --- | --- | --- | --- | --- |
| S/NO | AGE | SEX | BMI | GROUP | SURGICAL DURATION (min) | FOLLOW-UP PERIOD (Months) | COMPLICATIONS | NEED FOR REOPERATION | HOSPITALIZATION PERIOD (Days) | TOTAL COST(Yuan) | PREOPS LEG VAS SCORE | LEG PAIN VAS SCORE AT DISCHARGE | LEG POST OPS 6 MONTH VAS SCORE | LEG PAIN POST OPS 12 MONTHS VAS SCORE | PATIENT SATISFACTION | AFFECTED INTERVERTEBRAL DISC | Admission number | BLEEDING (ml) | PREOPS BP VAS SCORE | BP VAS SCORE AT DISCHARGE | BP POST OPS 6 MONTH VAS SCORE | B P POST OPS 12 MONTHS VAS SCORE | PREOPS JOA SCORE | JOA SCORE AT DISCHARGE |
| 1 | 75 | Male | 22.6 | Not sutured | 95 | 24 | No complication | No need | 9 | 23,173.79 | 7.00 | 3 | 2 | 1 | Highly satisfied | L5/S1 | 1808408 | 40 | 6.00 | 3 | 2 | 1 | 11 | 23 |
| 2 | 38 | Male | 25.4 | Not sutured | 150 | 24 | No complication | No need | 9 | 22,252.52 | 5.00 | 2 | 1 | 0 | Highly satisfied | L5/S1 | 1808221 | 50 | 7.00 | 2 | 1 | 0 | 13 | 25 |
| 3 | 40 | Male | 26 | Not sutured | 90 | 25 | No complication | No need | 11 | 21,012.76 | 6.00 | 2 | 1 | 0 | Highly satisfied | L5/S1 | 1800534 | 20 | 5.00 | 2 | 1 | 0 | 9 | 21 |
| 4 | 78 | Male | 24.4 | Not sutured | 80 | 25 | No complication | No need | 10 | 23,981.31 | 6.00 | 3 | 2 | 0 | Satisfied | L3/L4 | 1801619 | 20 | 5.00 | 3 | 2 | 0 | 10 | 22 |
| 5 | 76 | Female | 22.7 | Not sutured | 100 | 24 | No complication | No need | 7 | 19,517.63 | 7.00 | 4 | 2 | 1 | Highly satisfied | L4/L5 | 1802273 | 20 | 6.00 | 4 | 2 | 1 | 15 | 22 |
| 6 | 65 | Male | 29.4 | Not sutured | 155 | 25 | No complication | No need | 10 | 24,164.55 | 8.00 | 3 | 1 | 0 | Satisfied | L4/L5 | 1803175 | 20 | 7.00 | 3 | 1 | 0 | 12 | 19 |
| 7 | 62 | Male | 21.5 | Not sutured | 90 | 25 | No complication | No need | 8 | 22,378.16 | 5.00 | 2 | 1 | 1 | Highly satisfied | L4/L5 | 1803872 | 50 | 5.00 | 2 | 1 | 1 | 10 | 18 |
| 8 | 61 | Female | 25.2 | Not sutured | 80 | 24 | No complication | No need | 9 | 22,249.26 | 6.00 | 3 | 1 | 1 | Highly satisfied | L5/S1 | 1805557 | 20 | 5.00 | 3 | 1 | 1 | 12 | 20 |
| 9 | 65 | Female | 23.6 | Not sutured | 135 | 24 | No complication | No need | 8 | 21,170.31 | 5.00 | 2 | 1 | 1 | Satisfied | L5/S1 | 1805853 | 20 | 5.00 | 2 | 1 | 1 | 10 | 18 |
| 10 | 58 | Male | 24.3 | Not sutured | 90 | 24 | No complication | No need | 10 | 22,913.47 | 4.00 | 2 | 1 | 0 | Highly satisfied | L4/L5 | 1806022 | 20 | 4.00 | 2 | 1 | 0 | 14 | 17 |
| 11 | 47 | Male | 31.1 | Not sutured | 115 | 24 | No complication | No need | 8 | 21,377.51 | 6.00 | 2 | 1 | 0 | Satisfied | L4/L5 | 1806528 | 30 | 6.00 | 2 | 1 | 0 | 14 | 18 |
| 12 | 33 | Male | 22.3 | Not sutured | 140 | 24 | No complication | No need | 7 | 20,975.68 | 5.00 | 3 | 2 | 0 | Highly satisfied | L5/S1 | 1806754 | 40 | 7.00 | 3 | 2 | 0 | 8 | 25 |
| 13 | 38 | Male | 22.9 | Not sutured | 150 | 24 | No complication | No need | 9 | 22,252.52 | 5.00 | 2 | 1 | 0 | Highly satisfied | L5/S1 | 1808221 | 50 | 6.00 | 2 | 1 | 0 | 12 | 26 |
| 14 | 75 | Male | 25.8 | Not sutured | 95 | 26 | No complication | No need | 9 | 23,173.79 | 6.00 | 3 | 2 | 1 | Highly satisfied | L4/L5 | 1808408 | 20 | 5.00 | 3 | 2 | 1 | 11 | 27 |
| 15 | 63 | Male | 19.9 | Not sutured | 139 | 25 | No complication | No need | 10 | 27,117.29 | 6.00 | 3 | 2 | 1 | Highly satisfied | L4/L5 | 1809350 | 30 | 5.00 | 3 | 2 | 1 | 13 | 22 |
| 16 | 65 | Female | 28.9 | Not sutured | 56 | 24 | No complication | No need | 8 | 23,627.48 | 5.00 | 2 | 1 | 0 | Satisfied | L5/S1 | 1810235 | 20 | 4.00 | 2 | 1 | 0 | 10 | 23 |
| 17 | 62 | Female | 25.3 | Not sutured | 95 | 27 | Relapse | Reoperated | 10 | 34,733.00 | 5.00 | 4 | 2 | 1 | Satisfied | L4/L5 | 1810476 | 20 | 4.00 | 4 | 2 | 1 | 8 | 24 |
| 18 | 59 | Female | 27.8 | Not sutured | 105 | 28 | No complication | No need | 8 | 22,865.16 | 7.00 | 2 | 1 | 0 | Highly satisfied | L5/S1 | 1810506 | 10 | 6.00 | 2 | 1 | 0 | 12 | 21 |
| 19 | 60 | Male | 26 | Not sutured | 70 | 27 | Relapse | Reoperated | 5 | 22,366.15 | 8.00 | 4 | 3 | 2 | Satisfied | L4/L5 | 1810669 | 20 | 6.00 | 4 | 3 | 2 | 9 | 23 |
| 20 | 56 | Male | 24.2 | Not sutured | 85 | 26 | No complication | No need | 8 | 22,367.51 | 7.00 | 2 | 1 | 0 | Highly satisfied | L4/L5 | 1811325 | 20 | 6.00 | 2 | 1 | 0 | 12 | 26 |
| 21 | 57 | Female | 23.2 | Not sutured | 100 | 24 | No complication | No need | 10 | 23,836.73 | 5.00 | 3 | 2 | 1 | Highly satisfied | L5/S1 | 1811369 | 20 | 4.00 | 3 | 2 | 1 | 13 | 25 |
| 22 | 66 | Male | 27.7 | Not sutured | 90 | 29 | No complication | No need | 8 | 21,487.66 | 5.00 | 3 | 2 | 1 | Highly satisfied | L5/S1 | 1811982 | 30 | 4.00 | 3 | 2 | 1 | 9 | 25 |
| 23 | 59 | Female | 22.6 | Not sutured | 80 | 24 | No complication | No need | 8 | 22,999.49 | 5.00 | 3 | 2 | 0 | Highly satisfied | L4/L5 | 1812127 | 20 | 5.00 | 3 | 2 | 0 | 10 | 20 |
| 24 | 56 | Male | 26.8 | Not sutured | 90 | 25 | No complication | No need | 9 | 22,312.36 | 4.00 | 2 | 1 | 0 | Highly satisfied | L5/S1 | 1812208 | 60 | 4.00 | 2 | 1 | 0 | 6 | 20 |
| 25 | 65 | Female | 22.8 | Not sutured | 50 | 27 | No complication | No need | 7 | 21,060.29 | 4.00 | 2 | 2 | 0 | Highly satisfied | L5/S1 | 1813134 | 20 | 4.00 | 2 | 2 | 0 | 7 | 21 |
| 26 | 58 | Female | 20.9 | Not sutured | 90 | 25 | No complication | No need | 11 | 24,507.62 | 6.00 | 3 | 1 | 0 | Satisfied | L4/L5 | 1813542 | 20 | 5.00 | 3 | 1 | 0 | 10 | 23 |
| 27 | 38 | Female | 25 | Not sutured | 80 | 27 | No complication | No need | 12 | 20,191.11 | 7.00 | 4 | 3 | 1 | Highly satisfied | L5/S1 | 1813604 | 10 | 6.00 | 4 | 3 | 1 | 8 | 25 |
| 28 | 42 | Female | 23.9 | Not sutured | 65 | 26 | No complication | No need | 8 | 20,116.68 | 6.00 | 3 | 1 | 1 | Highly satisfied | L4/L5 | 1817303 | 10 | 5.00 | 3 | 1 | 1 | 7 | 21 |
| 29 | 31 | Female | 26 | Not sutured | 105 | 24 | Post operative bleeding | No need | 10 | 23,845.68 | 5.00 | 2 | 2 | 1 | Satisfied | L4/L5 | 1818015 | 20 | 4.00 | 2 | 2 | 1 | 9 | 23 |
| 30 | 65 | Male | 23.8 | Not sutured | 95 | 25 | No complication | No need | 8 | 24,297.77 | 6.00 | 3 | 1 | 0 | Highly satisfied | L4/L5 | 1818162 | 20 | 6.00 | 3 | 1 | 0 | 6 | 22 |
| 31 | 39 | Male | 20.9 | Sutured | 145 | 26 | No complication | No need | 11 | 23,539.11 | 6.00 | 3 | 1 | 0 | Highly satisfied | L5/S1 | 1818174 | 20 | 5.00 | 3 | 1 | 0 | 9 | 22 |
| 32 | 74 | Male | 19.7 | Not sutured | 105 | 25 | No complication | No need | 6 | 17,304.17 | 5.00 | 2 | 0 | 0 | Highly satisfied | L4/L5 | 1819512 | 20 | 4.00 | 2 | 0 | 0 | 10 | 21 |
| 33 | 69 | Female | 23.4 | Sutured | 149 | 24 | No complication | No need | 12 | 25,400.31 | 5.00 | 2 | 0 | 0 | Highly satisfied | L4/L5 | 1819882 | 20 | 4.00 | 2 | 0 | 0 | 10 | 20 |
| 34 | 68 | Female | 23.4 | Not sutured | 85 | 27 | No complication | No need | 9 | 22,021.02 | 5.00 | 3 | 1 | 0 | Highly satisfied | L4/L5 | 1819925 | 20 | 4.00 | 3 | 1 | 0 | 12 | 21 |
| 35 | 52 | Female | 21.5 | Not sutured | 115 | 24 | No complication | No need | 9 | 23,088.40 | 6.00 | 3 | 1 | 0 | Satisfied | L5/S1 | 1820588 | 30 | 5.00 | 3 | 1 | 0 | 11 | 18 |
| 36 | 78 | Female | 25.8 | Sutured | 221 | 28 | No complication | No need | 10 | 43,005.19 | 6.00 | 2 | 0 | 0 | Satisfied | L4/L5 | 1821313 | 50 | 5.00 | 2 | 0 | 0 | 7 | 19 |
| 37 | 84 | male | 21.9 | Sutured | 113 | 26 | No complication | No need | 10 | 26,028.06 | 6.00 | 2 | 0 | 0 | Highly satisfied | L5/S1 | 1821440 | 20 | 6.00 | 2 | 0 | 0 | 8 | 20 |
| 38 | 75 | Female | 25.6 | sutured | 151 | 24 | No complication | No need | 7 | 25,356.45 | 5.00 | 3 | 1 | 0 | Highly satisfied | L5/S1 | 1822396 | 50 | 5.00 | 3 | 1 | 0 | 6 | 21 |
| 39 | 53 | Male | 21.5 | Not sutured | 138 | 25 | No complication | No need | 8 | 23,650.75 | 4.00 | 2 | 1 | 0 | Highly satisfied | L4/L5 | 1822411 | 10 | 4.00 | 2 | 1 | 0 | 9 | 23 |
| 40 | 78 | Female | 19.8 | Sutured | 170 | 26 | No complication | No need | 8 | 24,442.88 | 4.00 | 3 | 1 | 1 | Highly satisfied | L4/L5 | 1824861 | 30 | 4.00 | 3 | 1 | 1 | 9 | 24 |
| 41 | 71 | Male | 31.8 | Not sutured | 100 | 31 | No complication | No need | 8 | 24,263.44 | 6.00 | 3 | 0 | 0 | Highly satisfied | L4/L5 | 1825557 | 20 | 5.00 | 3 | 0 | 0 | 8 | 25 |
| 42 | 55 | Female | 22.3 | Sutured | 205 | 27 | No complication | No need | 8 | 22,736.68 | 5.00 | 3 | 0 | 0 | Highly satisfied | L4/L5 | 1825709 | 50 | 5.00 | 3 | 0 | 0 | 10 | 21 |
| 43 | 49 | Male | 24.2 | Not sutured | 80 | 27 | No complication | No need | 8 | 18,766.93 | 6.00 | 2 | 0 | 0 | Highly satisfied | L4/L5 | 1826105 | 10 | 5.00 | 2 | 0 | 0 | 12 | 22 |
| 44 | 48 | Female | 24.8 | Not sutured | 90 | 24 | No complication | No need | 7 | 21,592.68 | 6.00 | 3 | 1 | 1 | Highly satisfied | L5/S1 | 1826324 | 10 | 5.00 | 3 | 1 | 1 | 13 | 22 |
| 45 | 54 | Female | 21.4 | Sutured | 130 | 27 | No complication | No need | 7 | 21,599.93 | 5.00 | 3 | 1 | 0 | Highly satisfied | L4/L5 | 1826628 | 30 | 4.00 | 3 | 1 | 0 | 8 | 18 |
| 46 | 60 | Male | 26.3 | Not sutured | 110 | 25 | No complication | No need | 7 | 21,238.86 | 5.00 | 3 | 2 | 1 | Highly satisfied | L5/S1 | 1826767 | 50 | 4.00 | 3 | 2 | 1 | 9 | 19 |
| 47 | 65 | Female | 21.9 | Not sutured | 115 | 31 | No complication | No need | 6 | 21,145.34 | 6.00 | 2 | 1 | 0 | Satisfied | L5/S1 | 1827635 | 30 | 5.00 | 2 | 1 | 0 | 8 | 18 |
| 48 | 67 | Male | 23 | Not sutured | 110 | 32 | Relapse | Reoperated | 10 | 23,879.70 | 6.00 | 4 | 3 | 2 | Satisfied | L5/S1 | 1827981 | 10 | 5.00 | 4 | 3 | 2 | 10 | 24 |
| 49 | 57 | Female | 22.4 | Not sutured | 125 | 30 | No complication | No need | 11 | 23,242.65 | 5.00 | 3 | 1 | 0 | Highly satisfied | L5/S1 | 1828295 | 10 | 5.00 | 3 | 1 | 0 | 14 | 29 |
| 50 | 72 | male | 25.4 | Not sutured | 130 | 24 | No complication | No need | 11 | 23,284.76 | 6.00 | 3 | 0 | 0 | Highly satisfied | L4/L5 | 1828546 | 20 | 6.00 | 3 | 0 | 0 | 8 | 23 |
| 51 | 51 | Male | 28 | Not sutured | 110 | 26 | No complication | No need | 13 | 24,990.00 | 7.00 | 2 | 0 | 0 | Highly satisfied | L5/S1 | 1828997 | 20 | 6.00 | 2 | 0 | 0 | 10 | 25 |
| 52 | 63 | Male | 28.4 | Not sutured | 160 | 29 | Intra operative bleeding | No need | 23 | 32,118.38 | 7.00 | 3 | 1 | 0 | Highly satisfied | L4/L5 | 1829068 | 100 | 6.00 | 3 | 1 | 0 | 10 | 21 |
| 53 | 62 | Female | 29.3 | Not sutured | 85 | 27 | No complication | No need | 9 | 22,297.58 | 7.00 | 2 | 1 | 0 | Highly satisfied | L4/L5 | 1830600 | 20 | 6.00 | 2 | 1 | 0 | 12 | 22 |
| 54 | 64 | Female | 24.4 | Not sutured | 150 | 26 | No complication | No need | 17 | 32,287.10 | 7.00 | 3 | 0 | 0 | Highly satisfied | L4/L5 | 1831230 | 50 | 6.00 | 3 | 0 | 0 | 8 | 17 |
| 55 | 79 | Female | 23 | Not sutured | 120 | 25 | No complication | No need | 8 | 25,177.77 | 6.00 | 2 | 0 | 0 | Highly satisfied | L4/L5 | 1831287 | 20 | 5.00 | 2 | 0 | 0 | 9 | 17 |
| 56 | 58 | Female | 25.1 | Not sutured | 95 | 24 | No complication | No need | 9 | 22,775.34 | 6.00 | 3 | 1 | 1 | Highly satisfied | L4/L5 | 1832764 | 20 | 5.00 | 3 | 1 | 1 | 9 | 18 |
| 57 | 71 | Female | 23.4 | Not sutured | 90 | 26 | No complication | No need | 11 | 24,754.52 | 6.00 | 2 | 1 | 1 | Highly satisfied | L4/L5 | 1834325 | 20 | 5.00 | 2 | 1 | 1 | 10 | 27 |
| 58 | 85 | Male | 28.7 | Not sutured | 65 | 24 | No complication | No need | 22 | 43,882.93 | 6.00 | 3 | 2 | 1 | Satisfied | L4/L5 | 1835052 | 20 | 5.00 | 3 | 2 | 1 | 10 | 24 |
| 59 | 74 | Female | 20.4 | Not sutured | 80 | 27 | No complication | No need | 12 | 29,281.96 | 6.00 | 2 | 1 | 0 | Highly satisfied | L4/L5 | 1835387 | 50 | 6.00 | 2 | 1 | 0 | 13 | 25 |
| 60 | 64 | Female | 19.2 | Not sutured | 120 | 29 | No complication | No need | 7 | 19,539.87 | 7.00 | 3 | 1 | 1 | Highly satisfied | L5/S1 | 1836495 | 20 | 6.00 | 3 | 1 | 1 | 12 | 23 |
| 61 | 32 | Male | 21.2 | Not sutured | 60 | 27 | No complication | No need | 6 | 20,193.91 | 8.00 | 2 | 0 | 0 | Highly satisfied | L3/L4 | 1837049 | 20 | 7.00 | 2 | 0 | 0 | 13 | 23 |
| 62 | 69 | Male | 29.1 | Not sutured | 65 | 24 | No complication | No need | 11 | 24,458.89 | 7.00 | 3 | 0 | 0 | Highly satisfied | L4/L5 | 1837567 | 50 | 6.00 | 3 | 0 | 0 | 14 | 22 |
| 63 | 48 | female | 21.8 | Not sutured | 90 | 30 | No complication | No need | 10 | 22,285.73 | 6.00 | 2 | 1 | 0 | Highly satisfied | L5/S1 | 1837610 | 10 | 5.00 | 2 | 1 | 0 | 9 | 21 |
| 64 | 58 | Male | 22.6 | Not sutured | 90 | 25 | No complication | No need | 7 | 23,462.56 | 7.00 | 3 | 1 | 0 | Highly satisfied | L4/L5 | 1839585 | 50 | 6.00 | 3 | 1 | 0 | 8 | 20 |
| 65 | 66 | Female | 22.5 | Not sutured | 120 | 24 | Intraoperetive bleeding | No need | 7 | 23,869.81 | 8.00 | 2 | 1 | 0 | Satisfied | L4/L5 | 1839729 | 100 | 7.00 | 2 | 1 | 0 | 9 | 27 |
| 66 | 35 | Male | 20.2 | Not sutured | 60 | 30 | No complication | No need | 9 | 20,445.08 | 6.00 | 3 | 0 | 0 | Highly satisfied | L4/L5 | 1840355 | 20 | 5.00 | 3 | 0 | 0 | 12 | 21 |
| 67 | 45 | Male | 24.2 | Not sutured | 80 | 33 | No complication | No need | 5 | 19,691.15 | 6.00 | 3 | 0 | 0 | Highly satisfied | L5/S1 | 1840439 | 20 | 5.00 | 3 | 0 | 0 | 12 | 22 |
| 68 | 60 | Female | 20.2 | Not sutured | 55 | 24 | No complication | No need | 10 | 23,430.09 | 7.00 | 2 | 1 | 0 | Highly satisfied | L5/S1 | 1840620 | 20 | 6.00 | 2 | 1 | 0 | 11 | 20 |
| 69 | 66 | Female | 21.6 | Not sutured | 60 | 28 | No complication | No need | 7 | 14,092.79 | 6.00 | 3 | 1 | 0 | Highly satisfied | L4/L5 | 1840682 | 50 | 5.00 | 3 | 1 | 0 | 10 | 24 |
| 70 | 57 | Male | 22 | Not sutured | 80 | 29 | No complication | No need | 5 | 20,794.72 | 7.00 | 3 | 1 | 1 | Highly satisfied | L5/S1 | 1840737 | 20 | 6.00 | 3 | 1 | 1 | 15 | 25 |
| 71 | 69 | Male | 22.8 | Not sutured | 76 | 32 | No complication | No need | 14 | 20,570.31 | 6.00 | 3 | 2 | 1 | Highly satisfied | L5/S1 | 1842686 | 30 | 5.00 | 3 | 2 | 1 | 14 | 23 |
| 72 | 72 | Female | 28.4 | Not sutured | 80 | 29 | No complication | No need | 15 | 23,723.23 | 6.00 | 2 | 1 | 0 | Satisfied | L4/L5 | 1842889 | 50 | 5.00 | 2 | 1 | 0 | 12 | 26 |
| 73 | 33 | Male | 24.4 | Not sutured | 90 | 31 | No complication | No need | 13 | 23,911.22 | 7.00 | 2 | 1 | 0 | Highly satisfied | L4/L5 | 1843580 | 50 | 6.00 | 2 | 1 | 0 | 12 | 27 |
| 74 | 68 | Female | 19 | Sutured | 124 | 27 | No complication | No need | 11 | 24,447.03 | 8.00 | 3 | 2 | 1 | Highly satisfied | L4/L5 | 1843795 | 30 | 7.00 | 3 | 2 | 1 | 14 | 21 |
| 75 | 49 | Male | 25 | Not sutured | 75 | 25 | No complication | No need | 9 | 23,428.57 | 7.00 | 3 | 2 | 0 | Highly satisfied | L4/L5 | 1843975 | 50 | 6.00 | 3 | 2 | 0 | 12 | 23 |
| 76 | 66 | Female | 22.2 | Not sutured | 65 | 26 | No complication | No need | 8 | 23,161.98 | 8.00 | 3 | 1 | 0 | Highly satisfied | L4/L5 | 1844430 | 20 | 7.00 | 3 | 1 | 0 | 8 | 22 |
| 77 | 77 | Female | 31.2 | Not sutured | 55 | 30 | No complication | No need | 8 | 23,753.06 | 6.00 | 2 | 2 | 1 | Highly satisfied | L4/L5 | 1844866 | 30 | 5.00 | 2 | 2 | 1 | 6 | 17 |
| 78 | 72 | Female | 22.9 | Sutured | 120 | 28 | No complication | No need | 11 | 24,255.92 | 6.00 | 3 | 2 | 0 | Highly satisfied | L4/L5 | 1846541 | 50 | 5.00 | 3 | 2 | 0 | 5 | 18 |
| 79 | 71 | Female | 20.5 | Not sutured | 83 | 25 | No complication | No need | 6 | 18,717.23 | 6.00 | 3 | 2 | 1 | Highly satisfied | L4/L5 | 1847730 | 30 | 5.00 | 3 | 2 | 1 | 7 | 19 |
| 80 | 63 | Female | 28.9 | Not sutured | 70 | 29 | No complication | No need | 11 | 23,246.28 | 7.00 | 2 | 1 | 0 | Highly satisfied | L3/L4 | 1847973 | 20 | 6.00 | 2 | 1 | 0 | 5 | 18 |
| 81 | 76 | Female | 23.3 | Not sutured | 116 | 26 | No complication | No need | 9 | 23,134.86 | 6.00 | 2 | 1 | 0 | Highly satisfied | L4/L5 | 1848883 | 10 | 5.00 | 2 | 1 | 0 | 6 | 21 |
| 82 | 55 | Male | 25.6 | Not sutured | 125 | 24 | No complication | No need | 9 | 19,123.76 | 5.00 | 3 | 1 | 0 | Highly satisfied | L4/L5 | 1850573 | 100 | 5.00 | 3 | 1 | 0 | 9 | 23 |
| 83 | 57 | Male | 30.2 | Not sutured | 50 | 28 | No complication | No need | 9 | 22,520.25 | 5.00 | 3 | 1 | 0 | Highly satisfied | L5/S1 | 1841017 | 30 | 5.00 | 3 | 1 | 0 | 8 | 26 |
| 84 | 59 | Male | 25.6 | Not sutured | 60 | 25 | No complication | No need | 9 | 22,363.05 | 5.00 | 2 | 1 | 0 | Highly satisfied | L4/L5 | 1851036 | 20 | 5.00 | 2 | 1 | 0 | 7 | 27 |
| 85 | 47 | Male | 23.2 | Not sutured | 70 | 31 | No complication | No need | 9 | 22,453.06 | 4.00 | 3 | 2 | 0 | Highly satisfied | L5/S1 | 1851143 | 30 | 4.00 | 3 | 2 | 0 | 6 | 28 |
| 86 | 75 | Male | 23.9 | Not sutured | 120 | 30 | No complication | No need | 7 | 22,551.84 | 6.00 | 2 | 1 | 0 | Highly satisfied | L5/S1 | 1852775 | 20 | 5.00 | 2 | 1 | 0 | 7 | 25 |
| 87 | 54 | Female | 30.5 | Not sutured | 60 | 29 | No complication | No need | 8 | 20,756.05 | 7.00 | 3 | 1 | 0 | Highly satisfied | L5/S1 | 1857701 | 30 | 6.00 | 3 | 1 | 0 | 8 | 21 |
| 88 | 50 | Male | 24.9 | Not sutured | 85 | 24 | No complication | No need | 8 | 20,960.01 | 5.00 | 2 | 0 | 0 | Highly satisfied | L4/L5 | 1857927 | 20 | 5.00 | 2 | 0 | 0 | 9 | 19 |
| 89 | 60 | Male | 21.6 | Not sutured | 62 | 25 | No complication | No need | 13 | 24,344.76 | 6.00 | 2 | 0 | 0 | Highly satisfied | L5/S1 | 1858986 | 20 | 5.00 | 2 | 0 | 0 | 5 | 18 |
| 90 | 74 | Female | 32.4 | Not sutured | 105 | 28 | No complication | No need | 8 | 19,527.90 | 5.00 | 3 | 0 | 0 | Highly satisfied | L5/S1 | 1859169 | 20 | 5.00 | 3 | 0 | 0 | 6 | 24 |
| 91 | 41 | Female | 20.5 | Not sutured | 140 | 27 | No complication | No need | 7 | 19,626 | 6 | 2 | 1 | 0 | Highly satisfied | L4/L5 | 1860054 | 50 | 6 | 2 | 1 | 0 | 6 | 26 |
| 92 | 68 | Female | 21.6 | Not sutured | 90 | 33 | No complication | No need | 9 | 15,530.72 | 5.00 | 3 | 2 | 1 | Highly satisfied | L5/S1 | 1860406 | 20 | 5.00 | 3 | 2 | 1 | 7 | 23 |
| 93 | 46 | Female | 21.2 | Not sutured | 95 | 25 | No complication | No need | 9 | 23,704.56 | 7.00 | 3 | 2 | 1 | Highly satisfied | L5/S1 | 1860610 | 100 | 6.00 | 3 | 2 | 1 | 8 | 19 |
| 94 | 51 | Male | 22.3 | Not sutured | 150 | 27 | No complication | No need | 7 | 20,591.11 | 8.00 | 3 | 1 | 0 | Highly satisfied | L5/S1 | 1860664 | 20 | 6.00 | 3 | 1 | 0 | 8 | 18 |
| 95 | 51 | Male | 25.4 | Sutured | 122 | 24 | No complication | No need | 6 | 21,066.93 | 5.00 | 2 | 0 | 0 | Highly satisfied | L4/L5 | 1861261 | 20 | 4.00 | 2 | 0 | 0 | 5 | 23 |
| 96 | 58 | Male | 28.7 | Not sutured | 85 | 27 | No complication | No need | 6 | 19,956.79 | 5.00 | 2 | 0 | 0 | Highly satisfied | L4/L5 | 1861422 | 20 | 4.00 | 2 | 0 | 0 | 5 | 25 |
| 97 | 39 | Male | 23.6 | Not sutured | 70 | 25 | No complication | No need | 6 | 18,752.46 | 7.00 | 3 | 1 | 0 | Highly satisfied | L4/L5 | 1862316 | 20 | 5.00 | 3 | 1 | 0 | 10 | 27 |
| 98 | 61 | Female | 23.4 | Not sutured | 85 | 28 | No complication | No need | 8 | 22,010.54 | 8.00 | 3 | 2 | 0 | Highly satisfied | L4/L5 | 1865313 | 20 | 6.00 | 3 | 2 | 0 | 9 | 28 |
| 99 | 55 | Male | 28.2 | Not sutured | 95 | 27 | No complication | No need | 8 | 23,523.72 | 6.00 | 3 | 1 | 0 | Satisfied | L4/L5 | 1865464 | 20 | 5.00 | 3 | 1 | 0 | 8 | 29 |
| 100 | 71 | Female | 29.3 | Sutured | 135 | 26 | No complication | No need | 8 | 23,839.72 | 7.00 | 2 | 0 | 0 | Highly satisfied | L4/L5 | 1866269 | 50 | 5.00 | 2 | 0 | 0 | 7 | 24 |
| 101 | 46 | Male | 23.7 | Not sutured | 95 | 30 | No complication | No need | 7 | 19,723.50 | 7.00 | 2 | 0 | 0 | Highly satisfied | L5/S1 | 1868235 | 20 | 5.00 | 2 | 0 | 0 | 9 | 24 |
| 102 | 58 | Female | 23.2 | Sutured | 80 | 26 | No complication | No need | 6 | 24,689.65 | 6.00 | 3 | 2 | 1 | Highly satisfied | L5/S1 | 1868785 | 20 | 5.00 | 3 | 2 | 1 | 10 | 19 |
| 103 | 65 | Female | 21.5 | Sutured | 90 | 25 | No complication | No need | 9 | 25,347.70 | 7.00 | 2 | 1 | 0 | Highly satisfied | L5/S1 | 1869389 | 20 | 6.00 | 2 | 1 | 0 | 9 | 18 |
| 104 | 58 | Female | 22 | Sutured | 170 | 27 | No complication | No need | 8 | 24,369.66 | 7.00 | 3 | 2 | 1 | Highly satisfied | L5/S1 | 1869810 | 50 | 5.00 | 3 | 2 | 1 | 7 | 17 |
| 105 | 29 | Male | 19.8 | Sutured | 155 | 27 | No complication | No need | 9 | 34,821.10 | 7.00 | 2 | 1 | 0 | Highly satisfied | L4/L5 | 1870292 | 20 | 6.00 | 2 | 1 | 0 | 7 | 18 |
| 106 | 77 | female | 24.7 | Sutured | 125 | 26 | No complication | No need | 10 | 28,834.10 | 6.00 | 2 | 0 | 0 | Highly satisfied | L4/L5 | 1870494 | 50 | 5.00 | 2 | 0 | 0 | 12 | 19 |
| 107 | 73 | Female | 21.3 | Not sutured | 65 | 24 | No complication | No need | 7 | 15,214.30 | 7.00 | 2 | 0 | 0 | Highly satisfied | L4/L5 | 1870699 | 50 | 6.00 | 2 | 0 | 0 | 12 | 23 |
| 108 | 43 | Female | 28 | Not sutured | 90 | 26 | No complication | No need | 8 | 21,807.86 | 8.00 | 3 | 0 | 0 | Highly satisfied | L5/S1 | 1871046 | 10 | 5.00 | 3 | 0 | 0 | 11 | 25 |
| 109 | 75 | Female | 24.9 | Not sutured | 70 | 27 | No complication | No need | 7 | 20,880.29 | 8.00 | 3 | 1 | 1 | Highly satisfied | L5/S1 | 1871954 | 20 | 5.00 | 3 | 1 | 1 | 8 | 27 |
| 110 | 58 | Female | 24.6 | Not sutured | 70 | 24 | No complication | No need | 9 | 20,548.21 | 7.00 | 2 | 2 | 1 | Highly satisfied | L5/S1 | 1872106 | 30 | 5.00 | 2 | 2 | 1 | 9 | 23 |
| 111 | 47 | Female | 20.1 | Not sutured | 130 | 26 | No complication | No need | 9 | 22,973.78 | 7.00 | 2 | 2 | 0 | Highly satisfied | L5/S1 | 1872719 | 100 | 6.00 | 2 | 2 | 0 | 10 | 24 |
| 112 | 65 | Male | 25.2 | Not sutured | 155 | 25 | No complication | No need | 9 | 23,115.93 | 8.00 | 3 | 1 | 0 | Highly satisfied | L5/S1 | 1872934 | 30 | 7.00 | 3 | 1 | 0 | 13 | 25 |
| 113 | 63 | Male | 23.5 | Not sutured | 120 | 31 | No complication | No need | 7 | 21,749.02 | 6.00 | 2 | 2 | 1 | Highly satisfied | L5/S1 | 1873433 | 50 | 5.00 | 2 | 2 | 1 | 11 | 20 |
| 114 | 73 | Male | 23.4 | Not sutured | 98 | 33 | No complication | No need | 11 | 24,408.08 | 6.00 | 3 | 1 | 0 | Highly satisfied | L4/L5 | 1874062 | 50 | 5.00 | 3 | 1 | 0 | 7 | 21 |
| 115 | 61 | Female | 23.5 | Not sutured | 102 | 34 | No complication | No need | 10 | 21,683.37 | 6.00 | 2 | 0 | 0 | Highly satisfied | L4/L5 | 1874309 | 10 | 6.00 | 2 | 0 | 0 | 8 | 26 |
| 116 | 71 | Female | 22.4 | Not sutured | 95 | 25 | No complication | No need | 11 | 22,391.38 | 7.00 | 2 | 0 | 0 | Highly satisfied | L4/L5 | 1874389 | 10 | 6.00 | 2 | 0 | 0 | 9 | 21 |
| 117 | 79 | Male | 23.4 | Not sutured | 100 | 26 | No complication | No need | 9 | 23,383.94 | 7.00 | 2 | 1 | 1 | Highly satisfied | L4/L5 | 1875462 | 30 | 6.00 | 2 | 1 | 1 | 10 | 23 |
| 118 | 52 | Male | 24.7 | Not sutured | 120 | 25 | No complication | No need | 7 | 18,477.45 | 6.00 | 3 | 1 | 1 | Highly satisfied | L5/S1 | 1876360 | 100 | 5.00 | 3 | 1 | 1 | 12 | 23 |
| 119 | 67 | Male | 21.9 | Not sutured | 120 | 27 | No complication | No need | 7 | 21,039.50 | 8.00 | 3 | 0 | 0 | Highly satisfied | L4/L5 | 1876365 | 20 | 5.00 | 3 | 0 | 0 | 7 | 21 |
| 120 | 53 | Male | 26 | Not sutured | 80 | 25 | No complication | No need | 7 | 20,051.42 | 5.00 | 3 | 1 | 0 | Highly satisfied | L4/L5 | 1876462 | 20 | 5.00 | 3 | 1 | 0 | 6 | 25 |
| 121 | 60 | Male | 21.2 | Not sutured | 80 | 24 | No complication | No need | 6 | 14,898.12 | 5.00 | 2 | 1 | 1 | Highly satisfied | L5/S1 | 1876556 | 50 | 5.00 | 2 | 1 | 1 | 5 | 19 |
| 122 | 69 | Male | 24.8 | Not sutured | 110 | 27 | No complication | No need | 7 | 22,297.34 | 6.00 | 3 | 1 | 0 | Satisfied | L4/L5 | 1878855 | 10 | 6.00 | 3 | 1 | 0 | 8 | 18 |
| 123 | 49 | Female | 24.6 | Not sutured | 90 | 26 | No complication | No need | 9 | 21,501.99 | 8.00 | 2 | 0 | 0 | Highly satisfied | L5/S1 | 1880374 | 20 | 5.00 | 2 | 0 | 0 | 9 | 23 |
| 124 | 57 | Female | 27.1 | Not sutured | 95 | 27 | No complication | No need | 9 | 21,040.80 | 7.00 | 2 | 1 | 0 | Highly satisfied | L4/L5 | 1880839 | 30 | 5.00 | 2 | 1 | 0 | 7 | 24 |
| 125 | 81 | Female | 25.2 | Not sutured | 95 | 26 | No complication | No need | 18 | 20,407.42 | 7.00 | 3 | 2 | 1 | Highly satisfied | L5/S1 | 1880859 | 20 | 5.00 | 3 | 2 | 1 | 8 | 26 |
| 126 | 75 | Female | 24.6 | Not sutured | 105 | 26 | No complication | No need | 8 | 20,714.10 | 6.00 | 2 | 1 | 1 | Highly satisfied | L4/L5 | 1881027 | 30 | 5.00 | 2 | 1 | 1 | 8 | 26 |
| 127 | 70 | Male | 22 | Not sutured | 90 | 29 | No complication | No need | 7 | 22,153.87 | 6.00 | 3 | 1 | 0 | Highly satisfied | L4/L5 | 1882053 | 20 | 5.00 | 3 | 1 | 0 | 6 | 25 |
| 128 | 57 | Female | 30.7 | Not sutured | 120 | 26 | Nerve compression by hematoma | Reoperated | 18 | 36,817.75 | 7.00 | 4 | 3 | 1 | Satisfied | L5/S1 | 1883014 | 50 | 6.00 | 4 | 3 | 1 | 7 | 28 |
| 129 | 49 | Male | 19.1 | Not sutured | 125 | 28 | No complication | No need | 9 | 22,002.50 | 7.00 | 3 | 1 | 0 | Highly satisfied | L4/L5 | 1883328 | 30 | 5.00 | 3 | 1 | 0 | 10 | 23 |
| 130 | 51 | Female | 28.6 | Sutured | 180 | 26 | No complication | No need | 8 | 30,520.67 | 6.00 | 3 | 2 | 1 | Highly satisfied | L4/L5 | 1883508 | 20 | 4.00 | 3 | 2 | 1 | 12 | 20 |
| 131 | 52 | Female | 28.2 | Sutured | 120 | 30 | No complication | No need | 11 | 30,679.07 | 6.00 | 3 | 2 | 1 | Highly satisfied | L4/L5 | 1883621 | 10 | 4.00 | 3 | 2 | 1 | 10 | 21 |
| 132 | 69 | Female | 30.9 | Not sutured | 90 | 27 | No complication | No need | 10 | 22,497.79 | 7.00 | 2 | 2 | 1 | Highly satisfied | L4/L5 | 1883650 | 20 | 5.00 | 2 | 2 | 1 | 9 | 28 |
| 133 | 55 | Male | 22.8 | Not sutured | 93 | 36 | No complication | No need | 15 | 23,429.31 | 6.00 | 2 | 1 | 0 | Highly satisfied | L4/L5 | 1883706 | 20 | 5.00 | 2 | 1 | 0 | 8 | 24 |
| 134 | 45 | Male | 32.3 | Not sutured | 60 | 35 | Relapse | Reoperated | 8 | 22,498.34 | 7.00 | 3 | 2 | 0 | Satisfied | L4/L5 | 1883785 | 50 | 6.00 | 3 | 2 | 0 | 8 | 24 |
| 135 | 37 | Male | 26.1 | Sutured | 115 | 28 | No complication | No need | 9 | 32,979.61 | 8.00 | 2 | 1 | 0 | Highly satisfied | L5/S1 | 1884448 | 50 | 6.00 | 2 | 1 | 0 | 13 | 21 |
| 136 | 41 | Male | 27 | Not sutured | 90 | 26 | No complication | No need | 10 | 22,702.27 | 7.00 | 2 | 0 | 0 | Highly satisfied | L5/S1 | 1884627 | 20 | 5.00 | 2 | 0 | 0 | 12 | 25 |
| 137 | 45 | Female | 24 | Not sutured | 75 | 29 | No complication | No need | 7 | 20,142.96 | 7.00 | 2 | 0 | 0 | Highly satisfied | L4/L5 | 1884957 | 10 | 5.00 | 2 | 0 | 0 | 8 | 27 |
| 137 | 61 | Female | 23.4 | Not sutured | 85 | 28 | No complication | No need | 9 | 20,940.70 | 8.00 | 2 | 0 | 0 | Highly satisfied | L4/L5 | 1885416 | 20 | 6.00 | 2 | 0 | 0 | 12 | 25 |
| 139 | 29 | Female | 29.4 | Not sutured | 100 | 36 | Relapse | Reoperated | 8 | 19,276.07 | 8.00 | 4 | 2 | 1 | Satisfied | L5/S1 | 1885438 | 30 | 5.00 | 4 | 2 | 1 | 7 | 21 |
| 140 | 36 | Male | 23.7 | Sutured | 150 | 27 | No complication | No need | 7 | 29,778.30 | 7.00 | 3 | 2 | 0 | Highly satisfied | L5/S1 | 1886413 | 20 | 6.00 | 3 | 2 | 0 | 9 | 24 |
| 141 | 64 | Female | 32 | Not sutured | 90 | 25 | No complication | No need | 9 | 22,301.16 | 7.00 | 3 | 1 | 0 | Highly satisfied | L4/L5 | 1888379 | 50 | 5.00 | 3 | 1 | 0 | 8 | 25 |
| 142 | 51 | Female | 14.2 | Not sutured | 70 | 27 | No complication | No need | 8 | 19,209.21 | 7.00 | 2 | 0 | 0 | Highly satisfied | L5/S1 | 1889166 | 10 | 6.00 | 2 | 0 | 0 | 9 | 26 |
| 142 | 71 | Female | 23.2 | Not sutured | 120 | 28 | No complication | No need | 7 | 20,867.56 | 7.00 | 3 | 1 | 0 | Highly satisfied | L4/L5 | 1889273 | 20 | 5.00 | 3 | 1 | 0 | 12 | 28 |
| 144 | 53 | Male | 24.6 | Sutured | 145 | 34 | No complication | No need | 7 | 30,173.74 | 8.00 | 2 | 0 | 0 | Highly satisfied | L5/S1 | 1890040 | 20 | 6.00 | 2 | 0 | 0 | 10 | 21 |
| 145 | 65 | male | 24.8 | Not sutured | 125 | 27 | Hemato nerve complression | Reoperated | 7 | 21,390.56 | 8.00 | 3 | 1 | 0 | Satisfied | L4/L5 | 1890185 | 20 | 5.00 | 3 | 1 | 0 | 11 | 21 |
| 146 | 43 | Female | 23.2 | Not sutured | 85 | 28 | No complication | No need | 7 | 19,783.51 | 7.00 | 3 | 1 | 0 | Highly satisfied | L5/S1 | 1890729 | 10 | 6.00 | 3 | 1 | 0 | 14 | 23 |
| 147 | 77 | Female | 23.2 | Not sutured | 70 | 26 | No complication | No need | 8 | 22,846.69 | 7.00 | 3 | 1 | 0 | Highly satisfied | L3/L4 | 1890857 | 50 | 6.00 | 3 | 1 | 0 | 8 | 24 |
| 148 | 49 | Female | 26.4 | Sutured | 195 | 27 | No complication | No need | 12 | 33,710.37 | 6.00 | 3 | 2 | 1 | Highly satisfied | L5/S1 | 1891016 | 20 | 5.00 | 3 | 2 | 1 | 9 | 27 |
| 149 | 51 | Female | 28.8 | Not sutured | 150 | 29 | No complication | No need | 7 | 20,931.54 | 5.00 | 3 | 1 | 0 | Highly satisfied | L4/L5 | 1891309 | 20 | 5.00 | 3 | 1 | 0 | 7 | 24 |
| 150 | 48 | male | 24.5 | Not sutured | 90 | 27 | No complication | No need | 8 | 21,543.60 | 5.00 | 2 | 0 | 0 | Highly satisfied | L5/S1 | 1891821 | 20 | 5.00 | 2 | 0 | 0 | 8 | 18 |
| 151 | 80 | Male | 20.8 | Sutured | 90 | 25 | No complication | No need | 10 | 38,488.04 | 7.00 | 3 | 0 | 0 | Highly satisfied | L5/S1 | 1892750 | 20 | 5.00 | 3 | 0 | 0 | 10 | 21 |
| 152 | 49 | Female | 19 | Sutured | 125 | 24 | No complication | No need | 9 | 29,308.30 | 6.00 | 3 | 1 | 0 | Highly satisfied | L5/S1 | 1893999 | 30 | 5.00 | 3 | 1 | 0 | 12 | 17 |
| 153 | 71 | Male | 22 | Not sutured | 120 | 28 | No complication | No need | 10 | 21,817.52 | 6.00 | 3 | 2 | 1 | Highly satisfied | L4/L5 | 1894063 | 20 | 6.00 | 3 | 2 | 1 | 13 | 18 |
| 154 | 48 | Male | 27.2 | Sutured | 115 | 30 | No complication | No need | 8 | 36,395.27 | 7.00 | 2 | 1 | 0 | Highly satisfied | L5/S1 | 1894246 | 20 | 5.00 | 2 | 1 | 0 | 12 | 16 |
| 155 | 52 | Female | 26.7 | Sutured | 75 | 24 | No complication | No need | 9 | 31,883.93 | 7.00 | 3 | 1 | 0 | Highly satisfied | L4/L5 | 1894408 | 20 | 6.00 | 3 | 1 | 0 | 13 | 17 |
| 156 | 63 | Male | 25.4 | Not sutured | 100 | 27 | No complication | No need | 8 | 21,002.58 | 8.00 | 2 | 2 | 0 | Highly satisfied | L4/L5 | 1894772 | 50 | 6.00 | 2 | 2 | 0 | 12 | 19 |
| 157 | 69 | Female | 24.1 | Sutured | 145 | 27 | No complication | No need | 11 | 22,179.94 | 7.00 | 3 | 1 | 0 | Highly satisfied | L4/L5 | 1894890 | 50 | 6.00 | 3 | 1 | 0 | 11 | 16 |
| 158 | 71 | Female | 23.7 | Not sutured | 100 | 28 | No complication | No need | 10 | 21,451.53 | 7.00 | 3 | 1 | 0 | Highly satisfied | L4/L5 | 1894950 | 50 | 5.00 | 3 | 1 | 0 | 8 | 18 |
| 159 | 55 | Female | 26.3 | Not sutured | 95 | 24 | No complication | No need | 9 | 19,922.70 | 5.00 | 3 | 1 | 0 | Highly satisfied | L4/L5 | 1895391 | 30 | 5.00 | 3 | 1 | 0 | 9 | 19 |
| 160 | 53 | Female | 22.9 | Sutured | 165 | 26 | Nerve compression by hematoma | Reoperated | 15 | 32,299.75 | 6.00 | 4 | 0 | 0 | Satisfied | L5/S1 | 1895714 | 50 | 6.00 | 4 | 0 | 0 | 7 | 20 |
| 161 | 60 | Female | 20.8 | Not sutured | 95 | 27 | No complication | No need | 9 | 20,244.72 | 7.00 | 3 | 0 | 0 | Highly satisfied | L4/L5 | 1896882 | 20 | 6.00 | 3 | 0 | 0 | 8 | 21 |
| 162 | 66 | Male | 24.8 | Not sutured | 85 | 26 | No complication | No need | 9 | 19,274.17 | 7.00 | 3 | 0 | 0 | Highly satisfied | L5/S1 | 1897731 | 20 | 6.00 | 3 | 0 | 0 | 9 | 17 |
| 163 | 48 | Male | 29.1 | Sutured | 70 | 28 | No complication | No need | 9 | 30,195.82 | 8.00 | 2 | 0 | 0 | Highly satisfied | L5/S1 | 1898463 | 10 | 5.00 | 2 | 0 | 0 | 7 | 19 |
| 164 | 45 | Male | 29.7 | Not sutured | 115 | 25 | No complication | No need | 7 | 18,270.54 | 6.00 | 3 | 1 | 0 | Highly satisfied | L5/S1 | 1899052 | 10 | 6.00 | 3 | 1 | 0 | 8 | 21 |
| 165 | 46 | Male | 30.9 | Sutured | 95 | 27 | No complication | No need | 8 | 29,451.28 | 6.00 | 3 | 2 | 1 | Highly satisfied | L4/L5 | 1899777 | 20 | 6.00 | 3 | 2 | 1 | 13 | 25 |
| 166 | 55 | Female | 27.3 | Sutured | 85 | 26 | No complication | No need | 7 | 30,311.65 | 8.00 | 2 | 0 | 0 | Highly satisfied | L4/L5 | 1899982 | 20 | 5.00 | 2 | 0 | 0 | 14 | 26 |
| 167 | 46 | Male | 24.6 | Sutured | 86 | 24 | No complication | No need | 10 | 33,088.05 | 6.00 | 3 | 2 | 1 | Highly satisfied | L4/L5 | 1900000 | 20 | 6.00 | 3 | 2 | 1 | 15 | 18 |
| 168 | 72 | Female | 19 | Sutured | 69 | 25 | No complication | No need | 13 | 40,729.74 | 5.00 | 2 | 1 | 0 | Highly satisfied | L5/S1 | 1900050 | 30 | 5.00 | 2 | 1 | 0 | 13 | 19 |
| 169 | 66 | Female | 29 | Sutured | 90 | 24 | No complication | No need | 8 | 31,866.49 | 6.00 | 3 | 1 | 0 | Highly satisfied | L4/L5 | 1900186 | 20 | 6.00 | 3 | 1 | 0 | 10 | 17 |
| 170 | 67 | Female | 26.7 | Not sutured | 95 | 25 | No complication | No need | 8 | 21,345.71 | 6.00 | 3 | 1 | 1 | Highly satisfied | L4/L5 | 1901898 | 20 | 6.00 | 3 | 1 | 1 | 9 | 18 |
| 171 | 71 | Female | 20.2 | Not sutured | 75 | 27 | No complication | No need | 8 | 22,616.57 | 7.00 | 3 | 0 | 0 | Highly satisfied | L4/L5 | 1902201 | 30 | 6.00 | 3 | 0 | 0 | 8 | 16 |
| 172 | 71 | Female | 23.6 | Sutured | 50 | 25 | No complication | No need | 9 | 29,525.96 | 7.00 | 3 | 0 | 0 | Highly satisfied | L5/S1 | 1903490 | 20 | 6.00 | 3 | 0 | 0 | 10 | 18 |
| 173 | 51 | Female | 28.1 | Sutured | 115 | 28 | No complication | No need | 8 | 30,319.97 | 7.00 | 2 | 1 | 1 | Highly satisfied | L5/S1 | 1904545 | 50 | 5.00 | 2 | 1 | 1 | 12 | 19 |
| 174 | 50 | Female | 20 | Sutured | 115 | 25 | No complication | No need | 9 | 34,875.31 | 7.00 | 2 | 1 | 0 | Highly satisfied | L4/L5 | 1904670 | 30 | 5.00 | 2 | 1 | 0 | 10 | 20 |
| 175 | 46 | Male | 25.5 | Sutured | 95 | 28 | No complication | No need | 10 | 27,614.80 | 8.00 | 2 | 0 | 0 | Highly satisfied | L5/S1 | 1904883 | 20 | 5.00 | 2 | 0 | 0 | 12 | 21 |
| 176 | 45 | Female | 26.7 | Not sutured | 150 | 26 | No complication | No need | 8 | 23,101.61 | 7.00 | 2 | 0 | 0 | Highly satisfied | L5/S1 | 1905685 | 50 | 5.00 | 2 | 0 | 0 | 9 | 17 |
| 177 | 74 | Male | 22.1 | Not sutured | 115 | 25 | No complication | No need | 11 | 26,663.60 | 8.00 | 3 | 0 | 0 | Highly satisfied | L5/S1 | 1907333 | 50 | 6.00 | 3 | 0 | 0 | 8 | 18 |
